# The Shortage of Medical Officers: An Appeal to the Colonies

**Published:** 1914-09-26

**Authors:** 


### THE EDITOR'S LETTER-BOX.

### The Shortage of Medical Officers: An Appeal to the Colonies.

*To the Editor of* The Hospital.

Sir,—The Tottenham Education Committee considered at its meeting last week the question of filling the vacancy of assistant school medical officer, but owing to the great dearth of medical men at this time the committee decided to defer the issue of advertisements at present.

In this connection may I mention a suggestion regarding the shortage of medical officers? Our Colonies have made splendid offers of fighting men and equipment for the Mother Country's need; why not invite them to send medical workers for the needs of the civil population in this country? It ought to be possible, where the qualifications of these volunteers are not legally registrable in this country, to issue a "certificate to practise" in a public institution—say, for the period of the war—always provided that the curriculum they have undergone is evidence of adequate training. Our American *confrères* would no doubt also lend a helping hand.

It may be urged that no lowering of the standard of training required for a medical qualification should be permitted. With that I agree. We are, however, dealing with exceptional circumstances which require immediate action if our sick persons are to have adequate treatment.—Yours faithfully,

Institutional Officer.

